# Syncytium-forming HSV-1 in cancer gene therapy: From molecular mechanisms to clinical translation

**DOI:** 10.1080/21505594.2026.2721822

**Published:** 2026-09-03

**Authors:** Xiangqian Zhao, Hucheng Zhang, Shuzhen Xu, Lingling Mei, Zhihua Feng, Dawei Wang, Weiwei Huang, Yangkun Shen

**Affiliations:** a Fujian Key Laboratory of Innate Immune Biology, Biomedical Research Center of South China, College of Life Science, Fujian Normal University, Fuzhou, China; b Central Laboratory, Fuzhou Hospital of Traditional Chinese Medicine Affiliated to Fujian University of Traditional Chinese Medicine, Fuzhou, China; c Clinical Oncology School of Fujian Medical University, Fujian Cancer Hospital, Fuzhou, Fujian Province, China

**Keywords:** Syncytium-forming, HSV-1, oncolytic virus, cancer gene therapy, immune modulation

## Abstract

Gene therapy has emerged as a promising strategy for cancer treatment, yet challenges in efficient gene delivery remain a major barrier. Herpes simplex virus type 1 (HSV-1), as an oncolytic virus, has garnered attention for its potential in cancer therapy due to its replicative capacity, large genomic payload, and relatively low toxicity. Notably, syncytium-forming HSV-1 (SF-HSV-1) not only exhibits enhanced and sustained antitumor efficacy but also triggers profound immune responses. However, the exact molecular mechanisms orchestrating HSV-1-induced syncytium formation, its resulting cytotoxicity, and its precise role in immune modulation remain incompletely understood. This review aims to provide an in-depth exploration of the mechanisms underlying HSV-1 syncytium formation and its therapeutic implications in cancer gene therapy.

## GRAPHICAL ABSTRACT

**Figure:**
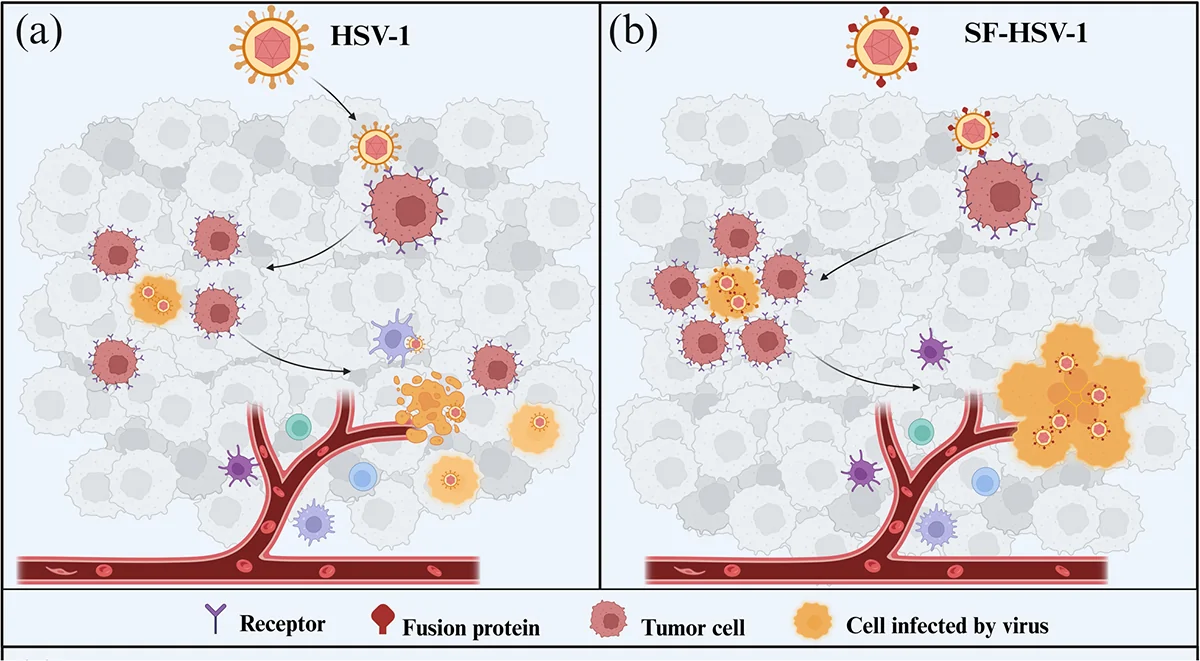

## Introduction

Gene therapy has shown considerable promise in cancer treatment by targeting genetic abnormalities within tumor cells. Traditional viral vectors, such as lentiviruses, retroviruses and adeno-associated viruses, deliver therapeutic genes to tumor cells, aiming to modulate oncogenes, inhibit angiogenesis, and activate tumor-suppressor pathways [1–5]. However, their limited gene-carrying capacity, risk of genomic integration, and inability to replicate within tumors restrict their clinical effectiveness [6–8].

In contrast, oncolytic viruses, particularly modified herpes simplex virus type 1 (HSV-1), have emerged as a compelling alternative due to their broad tumor lytic capacity, robust replication ability, substantial gene-carrying potential, and low toxicity. Conventional, non-syncytial HSV-1 platforms, such as Talimogene laherparepvec (T-VEC) and Teserpaturev (G47∆), are clinically established and approved for cancer treatment [9–12]. Despite their clinical maturity, their therapeutic efficacy remains frequently limited by insufficient tumor lysis, inadequate immune activation, and rapid clearance by preexisting antiviral antibodies in the tumor microenvironment [13,14].

To address these challenges, various strategies have been explored to increase the efficacy of oncolytic viruses [15]. One approach involves combining oncolytic virus therapy with other therapies such as radiotherapy, chemotherapy, and immunotherapy to achieve synergistic effects. Another strategy focuses on engineering oncolytic viruses to enable them better tumor-killing mechanisms. Interestingly, some viruses have the ability to induce host cells to form syncytia [16–23]. By driving the fusion of infected cells into multinucleated giant cells, SF-HSV-1 not only amplifies tumor cell killing but also profoundly enhances immune system responses by evading immune detection and boosting antigen presentation [24,25]. In sharp contrast to these mature, non-syncytial alternatives, syncytium-forming HSV-1 (SF-HSV-1) vectors remain a potent but strictly experimental platform; notably, Phase III clinical trial data demonstrate that RP1 exerts potent antitumor efficacy, and it also yields remarkable therapeutic effects against PD-1-resistant tumors [26,27]. Additionally, this strategy can significantly reduce the adverse effects of neutralizing antibodies, as the viral particles remain largely intact owing to their ability to disseminate intracellularly.

Syncytium formation is mediated by virus-expressed membrane fusion proteins, which enable fusion between the viral envelope and the host cell membrane and subsequently drive the fusion of multiple cells into syncytia. The mechanisms underlying syncytium formation involve complex conformational changes in fusion proteins, often triggered by specific cellular conditions or receptor interactions. Notably, fusion between the viral envelope and the host cell membrane is a prerequisite for syncytium formation. When these membrane fusion proteins are expressed on the surface of infected host cells, direct cell-to-cell fusion can occur, resulting in syncytium formation. In HSV-1, glycoproteins gB, gD, gH, and gL are essential for virus-mediated membrane fusion and syncytium formation, while mutations in genes such as gB, gK, UL20, and UL24 can further promote this phenomenon [28,29].

The mechanism of membrane fusion in SF-HSV-1 seems similar to that of conventional HSV-1. However, the underlying mechanism driving the syncytial phenotypic change remains unclear. The interactions between cell surface molecules and membrane rearrangement might be attributed to dysfunctions in viral fusion protein activity. Further investigations are needed to elucidate this phenomenon. Additionally, while SF-HSV-1 can evade immune recognition and activate antitumor responses through mechanisms such as the bystander effect and enhanced antigen presentation, the molecular details of how these effects are mediated remain unclear.

To provide a comprehensive roadmap from bench to bedside, this review is systematically organized into two major dimensions to bridge basic virology with therapeutic reality. We first delineate the precise molecular foundations and genetic determinants (such as *gB, gK*, and *UL20* mutations) that drive the transition from standard viral spread to hyperfusogenic syncytium formation. Progressing from these mechanisms, we critically evaluate the clinical translation of SF-HSV-1 platforms, detailing their therapeutic superiority in disrupting physical tumor barriers, driving robust immunogenic cell death (ICD), and navigating current translational hurdles such as off-target fusion risks and clinical safety profiles.

## Mechanism of HSV-1 intercellular transmission: cell-free and cell-to-cell spread

**Figure 1.**
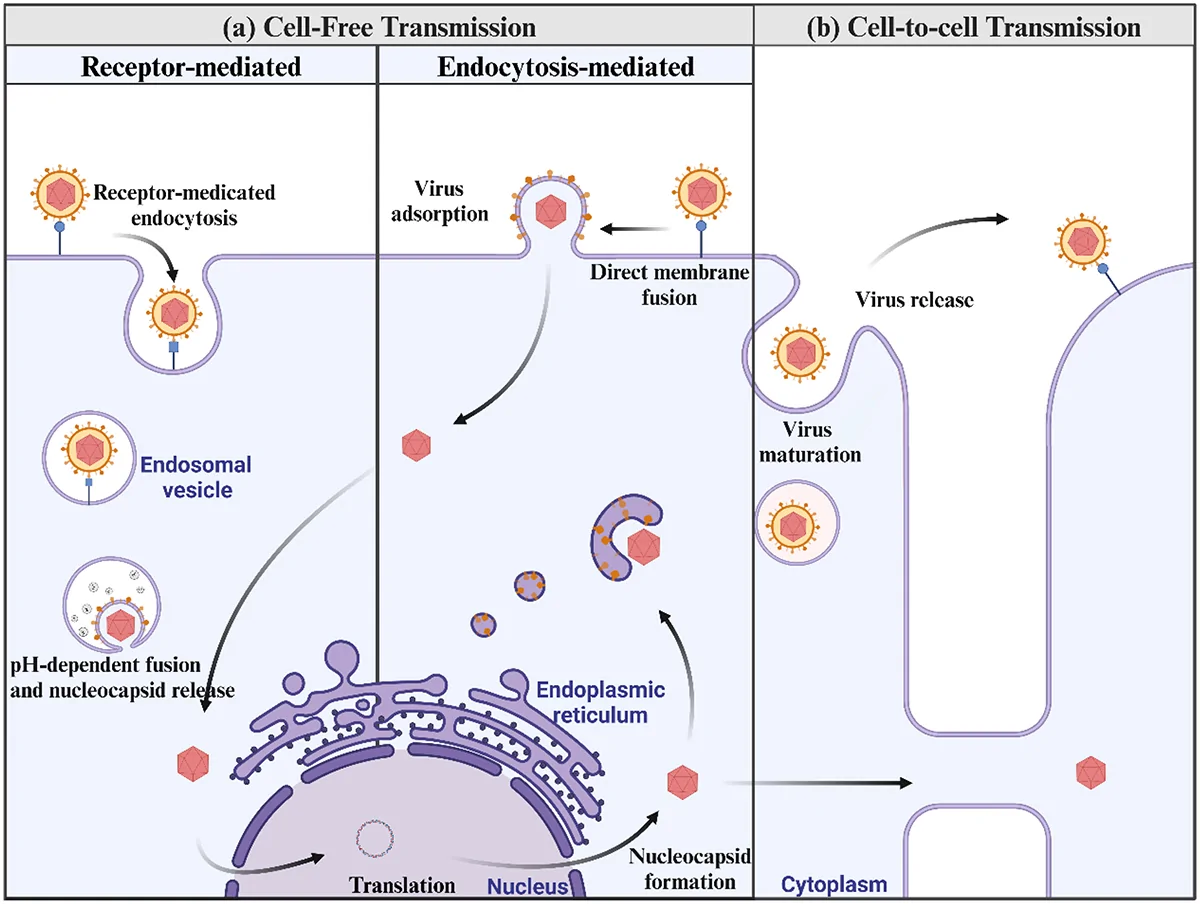
Mechanism of HSV-1 transmission. HSV-1 spreads between cells via two distinct mechanisms: cell-free transmission (a) and cell-to-cell transmission (b). Initial infection occurs through cell-free transmission, where the virus engages with cellular receptors via surface glycoproteins. Subsequent membrane fusion events involve either endocytosis followed by fusion within the host cell or direct fusion of the viral envelope with the host cell membrane. Once a host cell is infected, HSV-1 can spread rapidly between cells through multiple mechanisms. First, the virus is released extracellularly and enters neighboring cells. Alternatively, channels may open between adjacent cells, allowing viral nucleocapsids to rapidly transit from one cell to another.

Regardless of syncytium formation, the prerequisite for viral infection of cells is the occurrence of membrane fusion between the virus and the host cell. To understand HSV-1-mediated syncytium formation, it is necessary to understand the mechanisms of intercellular transmission of HSV-1. Existing data suggest that the intercellular transmission of HSV-1 involves two modes: cell-free transmission and cell-to-cell transmission [30] (Figure 1).

### Cell-free transmission

HSV-1 uses surface fusion proteins that directly attach to the cell surface of heparan sulfate (HS) and protein receptors, followed by membrane fusion and release of the viral capsid into the cell. The membrane fusion process of HSV-1 can occur in two ways: direct fusion of the viral envelope with the cell membrane or internalization of the virus via endocytosis, followed by membrane fusion within the cell [31]. HSV-1 infection involves a complex of viral glycoproteins (gB, gD, gH/gL) and host receptors that mediate membrane fusion [32,33]. These glycoproteins work in a sequential cascade to bind receptors and trigger fusion – a multi-step activation pathway robustly elucidated by foundational studies from Glorioso and colleagues [34]—ultimately releasing the viral nucleocapsid into the host cell.

HSV-1 glycoproteins gB and gC interact with heparan sulfate proteoglycans (HSPGs) on the host cell surface, facilitating adsorption [35]. Viral particles then move along the cell surface to allow gD to bind to specific receptors (nectin-1, nectin-2, HVEM), initiating irreversible attachment. Binding of gD to its receptor triggers a fusion signal, activating gH, which in turn activates gB. Conformational changes in gB lead to fusion of the viral envelope with the host cell membrane, releasing the viral nucleocapsid into the cytoplasm.

The endocytic pathway for cell entry is also important. HSV-1 can utilize both pathways for cell entry, depending on the type of host cell. It is suggested that the main pathway for HSV-1 entry into HeLa and CHO-K1 cell lines involves low pH-dependent endocytosis [36,37]. Although the specific role of low pH is unclear, endosomal acidification is necessary for capsid release (Figure 1(a)). In the subsequent membrane fusion process within endosomes, the low pH activates conformational changes in the fusion protein gB, which serves as a trigger for fusion [38]. While the precise conditions of the endocytic pathway in HSV infection have not been fully elucidated, the fusion mechanism undoubtedly plays a crucial role throughout this process [39].

### Cell-to-cell transmission

HSV-1 can spread rapidly between cells after successfully infecting the host cell by utilizing intercellular adhesion junctions. Before exiting the cell, the viral nucleocapsid first accumulates in the nucleus and then buds through the double nuclear membrane into the cytoplasm. The nucleocapsid subsequently acquires a tegument that is enveloped by either the trans-Golgi network (TGN) or endosomal membranes before it reaches the cell membrane. Ultimately, the enveloped membrane fuses with the cell membrane, releasing viral particles into the extracellular space [40] (Figure 1(a)).

However, viral particles are preferentially delivered to the lateral surface and cell junctions of polarized epithelial cells, where the envelope proteins gD, gE, and gI accumulate, rather than the apical surface, with gE/gI involved in this sorting process [41]. The gE/gI complex may facilitate the opening of intercellular channels (Figure 1(b)), allowing direct viral transit [42]. Moreover, the high expression of nectin-1 and nectin-2 receptors, which are involved in cell adhesion at intercellular junctions, enhances the intercellular spread of HSV-1 viruses [43,44]. While the exact biophysical mechanisms dictating this rapid transit remain partially obscured, it is widely hypothesized that this lateral spread limits extracellular viral exposure, thereby potentially shielding the virus from neutralizing antibodies [40]. However, further *in vivo* validation is required to establish definitive causality regarding immune evasion in complex tumor microenvironments. Ultimately, the profound syncytium formation induced by SF-HSV-1 represents the macroscopic extension of this intercellular propagation strategy.

## Mechanism of syncytium formation

**Figure 2.**
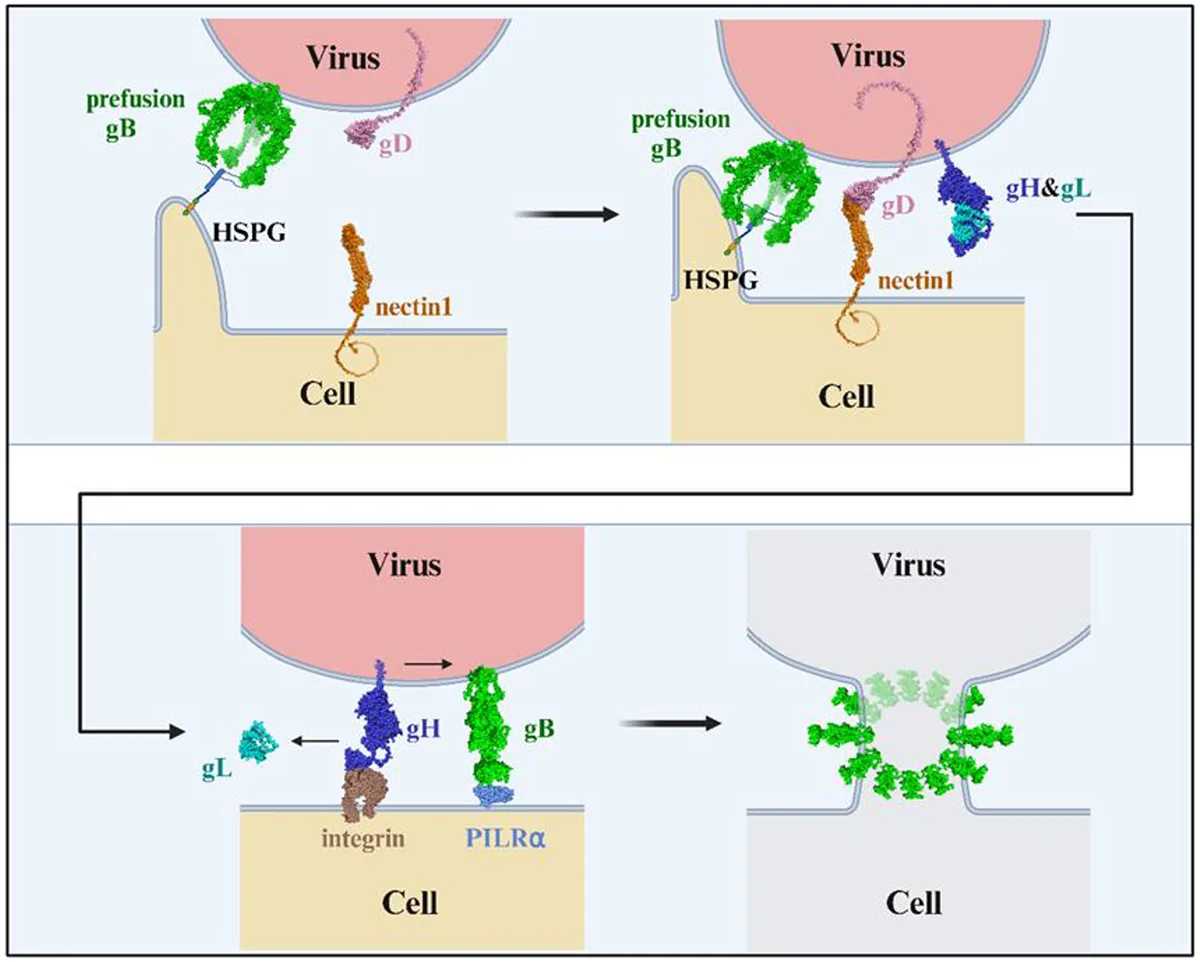
Model of the HSV-1 entry mechanism. Herpes simplex virus type 1 (HSV-1) fuses with the host cell membrane at the plasma membrane or endosomal membrane. The prefusion trimer of glycoprotein B (gB) (green) and glycoprotein C adsorb viral particles to the target cell surface through transient interactions with cell surface heparan sulfate proteoglycans (HSPGs). The viral particles then glide along the cell surface, allowing glycoprotein D (gD) (pink) to find its receptor, nectin-1 (orange). The receptor-bound gD transmits a fusion-promoting signal to the gH/gL complex (deep blue and light blue), which binds to integrins (brown). gL dissociates, and the activated gH transmits signals to gB, which binds to the receptor PILRα (blue), resulting in the activation of gB, ultimately leading to membrane fusion.

Blank et al. first reported the formation of syncytia in HSV-1-infected patients in 1951 [45]. Syncytia have long been regarded as a pathological indicator of α-herpesvirus infections, particularly in skin tissues and herpes simplex keratitis [46]. Although certain HSV mutants can form syncytia under physiological conditions, clinical isolates of HSV-1 rarely exhibit syncytial phenotypes [47,48]. However, mutants capable of syncytium formation can be easily isolated in vitro [46,49], typically resulting from one or a few viral gene mutations, primarily involving the *gB, gD, gK, UL20*, and *UL24* genes (Table 1). The other viral gene mutations related to the formation of syncytia are mostly associated with the “core fusion mechanism” (Figure 2).

**Table 1.** Summary of virus proteins and fusion machinery in HSV-1.

Gene	Protein	Function of the gene product	Effect on syncytium formation	Ref.
UL27	envelope glycoprotein B	Associate with membrane fusion	Point mutations cause extensive virus-induced cell fusion	[46,49,50]
UL53	envelope glycoprotein K	Glycoprotein required for efficient viral exocytosis	Display a reduced ability to block cell-cell fusion and endow fusogenicity,Point mutations cause virus-induced cell fusion	[51]
UL20	envelope protein UL20	UL20 is a viral envelope protein, hypothesized to prevent the fusion of infected cells with neighboring cells.	Point mutations cause virus-induced cell fusion	[49]
UL24	nuclear protein UL24	UL16 encodes a nuclear protein that is not essential for viral replication.	Point mutations cause virus-induced cell fusion	[52]
UL45	membrane protein UL45	It is involved in the gB-induced cell fusion process and is essential for viral replication.	The product of the UL45 gene is essential for gB-induced syncytium formation.	[53]
UL21	tegument protein UL21	UL21 is a viral envelope protein that can bind to microtubules. It is not required for viral replication in cultured cells.	The differences in syncytium phenotypes are essential.	[54]
UL16	tegument protein UL16	UL16 is a component of the viral envelope but is not essential for viral replication.	Different syncytium mutant strains have varying requirements for gE, gI, and UL16.	[49]
US8	envelope glycoprotein E	gI forms a heterodimer with gE, assisting the virus in entering cells. While it is dispensable for viral proliferation in differentiated cells, it is crucial in undifferentiated cells.		
US7	envelope glycoprotein I			

### Mechanisms of syncytium formation mediated by gB, gK and UL20 mutations

gB is a class III viral fusion protein. It regulates membrane fusion in four stages: tight binding, hemifusion, small fusion pore formation, and large fusion pore formation [50,55]. Studies have shown that the formation of fusion pores between the virus and host cell requires the participation of multiple gB trimers [56]. The fundamental genetic basis for this phenomenon was first established by the pioneering mapping studies by Bzik and colleagues who originally sequenced the complete gB gene [57], which set the foundation for subsequent work by Person and colleagues in pinpointing the precise nucleotide mutations dictating the syncytial (syn) phenotype [58,59]. Building upon these seminal works, it is now understood that all reported gB point mutations that induce syncytia formation are concentrated in the C-terminal domain (CTD) [46,49,50], and the deletion of 28 amino acids at the CTD can also trigger extensive fusion in Vero cells [60]. While the requirement of the gB CTD for syncytium formation is well-supported by extensive structural and mutational data, the exact downstream dynamic models – specifically how CTD conformational changes transmit signals to the ectodomain – remain largely speculative and warrant further investigation.

Lingen et al. [61,62] reported a strain that induces intercellular fusion during the viral binding-entry process, suggesting that point mutations in the CTD of gB may promote syncytium formation by altering the virus’s adsorption capability. However, simply influencing viral adsorption appears insufficient to fully explain syncytium formation, as promoting membrane fusion requires the formation of specific structures within both the intracellular and extracellular domains of gB. Silverman et al. [63] found that point mutations in the CTD of gB lead to conformational changes in the CTD, but do not alter the conformation of the extracellular domains or the stability of the trimer. Furthermore, in cells lacking gD and gH/gL, expression of gB with CTD point mutations alone does not induce syncytium formation [23]. These results suggest that the C-terminal domain (CTD) of glycoprotein B (gB) negatively modulates gB fusion activity via intrinsic conformational changes; mutations within the CTD attenuate this autoinhibitory effect, rendering gB competent to trigger excessive membrane fusion in response to subthreshold levels of activating signals, which ultimately culminates in syncytium formation.

The UL20 protein and gK play critical roles in cell fusion and syncytium formation in HSV-1. The foundational concept that specific viral glycoproteins inherently regulate – and can actively suppress – cell fusion was first revealed through the landmark discoveries by Spear and colleagues [64]. Decades of subsequent research have confirmed that both gK and UL20 are closely associated with gB-mediated virus-induced intercellular fusion. The expression of mutated UL20 and gK proteins alone is insufficient to induce syncytium formation, and this process fails in the absence of gB. However, syncytia associated with gK and UL20 mutations exhibit greater complexity, with mutation sites being irregularly distributed [65–68]. Experimental data have shown that UL20 deletion viruses suppress syncytial phenotypes induced by gB and gK mutations [69]. The precise regulatory roles of gK and UL20 remain a subject of debate, and their exact function remains controversial. Paradoxically, while some studies indicate that co-expression of gD, gH/gL, and gB is sufficient for fusion [70], conflicting reports exist stating that the addition of UL20 and gK can actually inhibit this process under certain conditions [71]. These conflicting findings suggest that the function of UL20/gK may be highly dependent on the specific viral strain background or the experimental assay used.

### Other genes involved in syncytium formation

*gD and UL24* are two other genes reported to cause syncytium formation due to point mutations. Rauch et al. identified two gD mutation sites (S140N and A185T) that induce syncytium formation, located in regions known to contain receptor-binding specificity determinants, suggesting that these mutations alter receptor affinity or specificity [72]. Pearson et al. and Bertrand et al. reported three mutations related to UL24 (G121A and E99A/K101A), a highly basic protein with complex regulation and late-expression leakage dynamics that plays a role in nucleolar remodeling during infection [52,73]. Other genes that affect syncytium formation have also been reported. For example, UL45 is essential for gB-induced syncytium formation [53]. Furthermore, SF-HSV-1 loses its syncytial phenotype in the absence of several interacting auxiliary proteins, including gE, gI, UL16, and UL21 [49,54].

Additionally, studies have shown that wild-type HSV-1 induces extensive syncytium formation following salubrinal treatment, suggesting that the target of salubrinal in host cells plays a pivotal role in syncytium formation [74]. Salubrinal-induced syncytium formation is reduced by inhibition or deletion of protein tyrosine phosphatase 1B (PTP1B) while HSV-1 titers remain unaffected, suggesting that PTP1B is another essential host factor involved in syncytium formation, but only intercellular spread is affected [75]. Although PTP1B has been shown not to be the target of Salubrinal, PTP1B is a host factor required for HSV-1-specific intercellular transmission and may serve as a potential therapeutic target for preventing HSV-1 reactivation.

Collectively, accumulating evidence indicates that syncytium-inducing mutations in gB are highly enriched within its cytoplasmic tail domain (CTD), whereas mutations with comparable fusogenic consequences in gK and UL20 are distributed throughout their coding regions without an obvious positional pattern. This distribution suggests that, in conventional HSV-1 infection, the gB CTD together with gK and UL20 may constitute a regulatory module that restrains inappropriate cell–cell fusion. Mutations affecting any component of this module may disrupt this inhibitory control, thereby lowering the threshold for membrane fusion and promoting syncytium formation. However, this model remains inferential and requires further validation through systematic mutagenesis, structural analyses, and comparative studies across different viral backgrounds and host cell types.

## Applications of SF-HSV-1 in cancer therapy

### Immune clearance evasion and antitumor efficacy of SF-HSV-1

**Figure 3.**
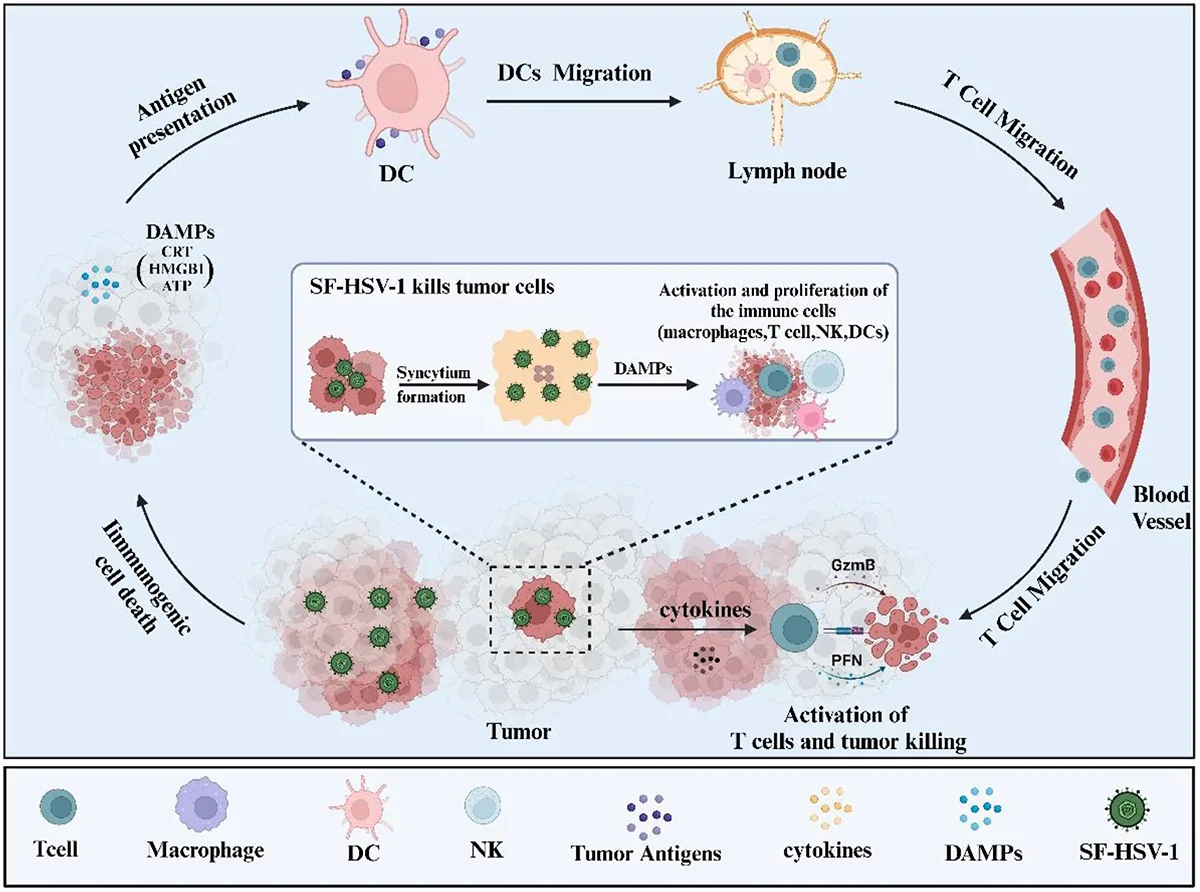
Immune activation effects of SF-HSV-1. The viral infection of tumor cells leads to the formation of syncytia, increasing cell lysis and promoting the immunogenic death of cancer cells, which activates the immune microenvironment. Tumor antigens are presented by dendritic cells (DCs) and enter the lymphatic system, where they activate lymphocytes and stimulate systemic antitumor immunity.

While wild-type viruses often face therapeutic limitations due to their reliance on vulnerable extracellular dissemination [75], the unique propagation dynamics of syncytial viruses offer a powerful alternative for cancer treatment. By capitalizing on direct membrane fusion, SF-HSV-1 operates as a highly efficient oncolytic vector, driving enhanced localized cytotoxicity and sustaining robust therapeutic activity even in immunologically hostile environments [76] (Figure 3).

Viruses can be categorized into two types on the basis of their ability to form syncytia through fusion proteins: naturally syncytial viruses and engineered syncytial viruses [77]. Several naturally syncytium-inducing viruses, including Newcastle disease virus [78,79], herpes simplex virus and respiratory syncytial virus [80,81], have been selected for oncolytic virus research [51,82,83]. The dense extracellular matrix (ECM) and elevated interstitial fluid pressure (IFP) characteristic of solid tumor microenvironments present severe physical bottlenecks for conventional, non-syncytial oncolytic viruses like Talimogene Laherparepvec (T-VEC). Released virions are frequently trapped locally and rapidly cleared by preexisting anti-HSV-1 neutralizing antibodies in the tumor interstitial fluid. By driving direct cell-to-cell plasma membrane fusion, SF-HSV-1 overcome these barriers and enable the virus to propagate laterally as an expanding multinucleated mass. This mechanism allows the therapeutic payload to traverse the rigid tumor stroma intracellularly, completely insulated from host neutralizing antibodies, thereby maximizing intratumoral dissemination even in poorly perfused tumor cores.

Preclinical studies have firmly demonstrated that this hyperfusogenic propagation effectively reduces tumor burden across various cancer models, such as glioblastoma-bearing mice where SF-HSV-1 significantly prolonged survival compared to controls. Luo et al. [51] reported that the oncolytic viruses derived from natural HSV-1 syncytia exhibit significantly increased cytotoxicity, with an increase of up to 59.63-fold. Subsequent experiments confirmed that these syncytial oncolytic viruses maintain relative safety.

The most extensively studied engineered SF-HSV-1 platforms utilize either the insertion of foreign fusogenic glycoproteins or the enhancement of endogenous viral fusion machinery. For instance, HSV-GALV combines an ICP34.5-deleted oncolytic virus backbone with a gibbon ape leukemia virus (GALV) fusion protein, demonstrating enhanced cytotoxicity both in vitro and in vivo due to increased membrane fusion capabilities [25,84–86]. To advance from laboratory models to clinical reality, these investigational SF-HSV-1 constructs must be contextualized against approved platforms. The most clinically advanced engineered SF-HSV-1 platform is RP1, which features an HSV-1 backbone modified to express a hyperfusogenic GALV envelope glycoprotein (GALV-GP-R) alongside GM-CSF. In the Phase II/III IGNYTE clinical trial evaluating RP1 in combination with nivolumab for patients with anti-PD-1-failed advanced melanoma, this platform demonstrated a highly compelling objective response rate (ORR). This starkly contrasts with the historical limitations of conventional T-VEC monotherapy in refractory patients, providing robust clinical evidence that a syncytial phenotype successfully revitalizes antitumor efficacy by overcoming therapeutic resistance. Additionally, one study designed an HSV encoding E-cadherin, which promotes the formation of syncytia to evade ICD-mediated clearance [87,88]. The expressed E-cadherin also disrupts the physical barriers within the tumor tissue, thereby enhancing viral spread within the tumor.

Despite multiple studies indicating that syncytium formation can improve antitumor immunity, research in this area remains limited and warrants further investigation as a focus for future studies.

### Immune activation effects of SF-HSV-1 in cancer therapy

HSV-1 triggers a complex immune response in the host, encompassing both innate and adaptive immunity [89]. For cancer therapy, like wild-type viruses, syncytial viruses effectively activate host cell innate immune responses, including the cGAS-STING, TLR-MyD88, and RIG-I-MDA5 pathways. This activation leads to the release of various cytokines and inflammatory mediators, stimulating broader activation of immune cells. Furthermore, syncytia may enhance the adaptive immune response through mechanisms such as improving antigen presentation and promoting bystander effects.

At the cell membrane, HSV-1 can activate TLR2, which interacts with MyD88, initiating the transcription of inflammatory cytokines and interferon-stimulated genes (ISGs) [90,91]. If HSV-1 enters the host cell via endocytosis, TLR3, TLR7/8, and TLR9 on the endosomal membrane are activated by dsRNA, ssRNA, and CpG DNA, inducing cytokine and ISG production via the NF-κB, IRF7, or IRF3 signaling pathways [92–94]. Additionally, viral RNAs can be recognized by cytosolic RIG-like receptors, which utilize MAVS as an adapter to activate IFN-I expression through NF-κB, and subsequent stimulation of cytokine and ISG production [95,96]. The DNA-sensing pathway also plays a significant role in the antiviral process. In the cytoplasm, cGAS binds viral dsDNA, leading to the activation of the interferon pathway [97–99]. Similarly, upon interaction with viral DNA, cytoplasmic IFI16, NLRP3, and AIM2 inflammasomes trigger the production of the proinflammatory cytokines IL-1β and IL-18 [100,101].

Adaptive immunity against HSV-1 develops over time and predominantly relies on CD8+ T cells while neutralizing antibodies play a relatively minor role. CD8+ T cells are recruited early during infection and are crucial for immune control. IFN-γ-producing CD8 T cells persist during latency but do not eliminate infection. Both CD4 and CD8 T cells prevent local mucosal replication of HSV-1 [102]. Antibody levels do not dictate infection outcomes, as seropositive mice show no significant impact on viral replication or latency, whereas B-cell-deficient mice are more susceptible to herpes encephalitis after ocular infection [103].

Crucially, the death of a massive multinucleated syncytium is immunologically distinct from standard individual cell apoptosis or standard lytic events. The synchronized collapse of these giant cells triggers ICD characterized by nuclear fusion, premature chromatin condensation, severe synchronous ATP depletion, and autophagic degradation accompanied by exosome release [104]. This macro-lysis prompts a massive, concentrated release of damage-associated molecular patterns (DAMPs) – such as calreticulin, HMGB1, and extracellular ATP – far exceeding the baseline levels induced by single-cell viral lysis [77,105]. Consequently, local dendritic cells (DCs) are recruited and activated at significantly higher efficiencies, markedly enhancing their capability to cross-present tumor-associated antigens (TAAs) and prime tumor-specific CD8+ T cells [16,104,106]. This unique ICD profile represents the primary analytical bridge explaining how SF-HSV-1 successfully converts a “cold” immunosuppressive tumor microenvironment into a “hot” highly immunoreactive hub capable of eliciting systemic antitumor immunity [77].

## Safety considerations and translational hurdles: balancing efficacy and toxicity

As SF-HSV-1 platforms transition from preclinical models to clinical trials, safety considerations must be elevated to the center of therapeutic design. The defining feature of SF-HSV-1 – its hyperfusogenic capability – acts as a double-edged sword. The delicate balance between maximizing intratumoral spread and minimizing off-target risks is the core translational bottleneck dictating the ultimate therapeutic index of these vectors.

### Off-target fusion and uncontrolled dissemination

While the lateral cell-to-cell spread effectively circumvents the dense tumor stroma, the nonspecific nature of this fusion cascade raises severe concerns regarding off-target tissue damage. This risk is particularly pronounced in immunocompromised oncology patients.

To mitigate this translational risk, viral tropism must be strictly and artificially constrained. Modern engineering strategies have demonstrated that utilizing tumor-specific promoters (e.g. TERT or survivin promoters) to drive essential viral genes or fusion cascades can strictly confine viral replication to malignant cells [51]. Additionally, incorporating microRNA (miRNA) target sequences – such as those for miR-122 (highly expressed in healthy hepatocytes) or miR-124 (expressed in normal neurons) – into the viral genome ensures rapid degradation of viral transcripts in normal tissues, effectively creating a physiological firewall against uncontrolled dissemination [107,108]. Furthermore, engineering the primary viral glycoproteins to selectively recognize tumor-associated antigens (e.g. HER2 or EGFR) has proven highly effective in retargeting the initial fusion event exclusively to tumor cells [109]. This fully retargeted platform design directly builds upon the pioneering strategies established by Glorioso et al., who elegantly demonstrated that oHSV vectors could be completely re-engineered to enter target cells solely via tumor-specific receptors (such as EGFR) while maintaining robust replication pipelines [110,111]. More recently, the integration of synthetic biology has introduced Boolean logic-gated circuits to SF-HSV-1 safety design. By employing strict “AND” gate configurations – where the hyperfusogenic cascade is exclusively triggered only if multiple distinct microenvironmental parameters (e.g. hypoxia-responsive elements combined with TERT activation) are simultaneously fulfilled – the probability of collateral fusion in normoxic, healthy tissues is mathematically minimized [112].

### Inflammatory toxicity and collateral damage

Beyond direct viral cytolysis, the profound immunogenic collapse of macroscopic syncytia poses a significant risk of severe inflammatory toxicity. While massive DAMP release is essential for breaking tumor tolerance, it simultaneously risks triggering hyper-inflammatory reactions akin to cytokine release syndrome (CRS). Extensive *in vivo* studies confirm that the unrestrained lysis of large syncytia is accompanied by dangerous systemic spikes in pro-inflammatory cytokines, including TNF-α, IL-6, and IL-1β [113].

This resultant cytokine storm can exacerbate collateral tissue damage and pose lethal systemic risks, particularly in patients with preexisting inflammatory comorbidities. Therefore, the next generation of SF-HSV-1 vectors must be armed not only with fusion engines but also with intrinsic immunoregulatory modules. Potential strategies to widen the therapeutic window include integrating genes that encode localized cytokine-scavenging receptors, or administering the virotherapy in strict coordination with transient systemic IL-6/IL-1β blockade therapies. To provide an absolute clinical guarantee against severe CRS or unmanageable dissemination, the latest generations of engineered vectors are incorporating ultra-fast fail-safe suicide switches, such as the inducible caspase-9 (iCasp9) system. Upon the administration of a bio-inert small-molecule dimerizer (e.g. AP1903), the entire syncytial mass is forced into rapid, synchronous apoptosis, instantly halting both viral replication and the propagating cytokine storm. This synthetic “kill switch” represents the ultimate therapeutic safety net, granting clinicians absolute pharmacological control over the powerful SF-HSV-1 platform [114]. Ultimately, realizing the full potential of SF-HSV-1 requires a carefully balanced design: the fusion cascade must be powerful enough to eradicate the tumor, yet sufficiently controlled to safeguard patient survival.

## Conclusion

SF-HSV-1 represents an important direction in the engineering of oncolytic HSV-1 platforms. Compared with conventional non-syncytial HSV-1 vectors, SF-HSV-1 promotes tumor therapy via enhanced local spread through cell-to-cell fusion, increased direct tumor cell killing, reduced exposure to extracellular neutralizing antibodies, and potential amplification of antitumor immune responses. These features are particularly relevant for solid tumors, where extracellular matrix barriers, heterogeneous vascularization, and immunosuppressive microenvironments often limit viral distribution and therapeutic efficacy. However, these advantages do not automatically translate into broad clinical benefit. The same fusogenic activity that may improve intratumoral spread also raises concerns about uncontrolled cell fusion, tissue damage, inflammatory toxicity, and tumor-type-dependent variability. Therefore, a deeper understanding of the fusion mechanism of SF-HSV-1 and its associated therapeutic safety risks is required. Its clinical efficacy will depend on careful vector design, appropriate tumor selection, and rigorous clinical validation.

A major unresolved question is how HSV-1 transitions from ordinary viral entry and local cell-to-cell spread to extensive syncytium formation. The available evidence indicates that HSV-1-induced fusion depends on the coordinated activities of gB, gD, gH/gL, and regulatory proteins such as gK, UL20, UL24, gE, gI, UL16, and UL21. Mutations in the cytoplasmic tail of gB and in genes such as *gK, UL20, gD*, and *UL24* can promote syncytial phenotypes, yet these mutations do not act in isolation. These mutations alone are insufficient to induce syncytium formation in the absence of other viral glycoproteins and cellular receptors. Moving forward, a more comprehensive mechanistic model is needed to elucidate how viral glycoproteins, capsid proteins, host receptors, membrane lipid composition, and intracellular signaling pathways act synergistically. This interplay ultimately determines whether an infection results in efficient viral entry, restricted cell-to-cell spread, or massive cell fusion. To address these questions, the field must transition from static *in vitro* structural snapshots to high-resolution spatial transcriptomics and *in situ* cryo-electron tomography (cryo-ET). This shift will enable the precise mapping of the actual structural transitions of fusion pores within complex living tissues [115].

Furthermore, the immunological consequences of SF-HSV-1 remain incompletely defined. Syncytium formation may enhance ICD, antigen release, dendritic cell cross-presentation, and CD8+ T-cell activation. It may also activate innate immune pathways such as cGAS-STING, TLR signaling, RIG-I/MDA5-MAVS signaling, and inflammasome-associated responses. These immune effects could support combination strategies with immune checkpoint inhibitors or other immunotherapies. However, immune activation is not uniformly beneficial. Excessive local or systemic inflammation may cause toxicity, and strong antiviral immunity may restrict viral persistence before a sufficient antitumor effect is achieved. In addition, the relative contribution of direct oncolysis, syncytial bystander killing, innate immune activation, and adaptive antitumor immunity remains difficult to separate. Future work should use comparative studies between matched syncytial and non-syncytial HSV-1 vectors to define which immune changes are truly caused by the syncytial phenotype rather than by viral replication, transgene expression, or tumor destruction in general.

Despite some progress in clinical translation, significant practical hurdles remain. First, safety remains a primary concern. Because cell fusion is inherently a membrane fusion process, off-target fusion between infected tumor cells and adjacent normal cells could theoretically cause tissue damage. This risk is particularly pronounced for tumors located near vital organs or in immunocompromised patients. Secondly, the manufacturing and regulatory evaluation of fusogenic vectors can be highly complex, particularly when multiple genetic modifications are employed to achieve tumor specificity, immunomodulation, and enhanced fusion capabilities.

**Figure 4.**
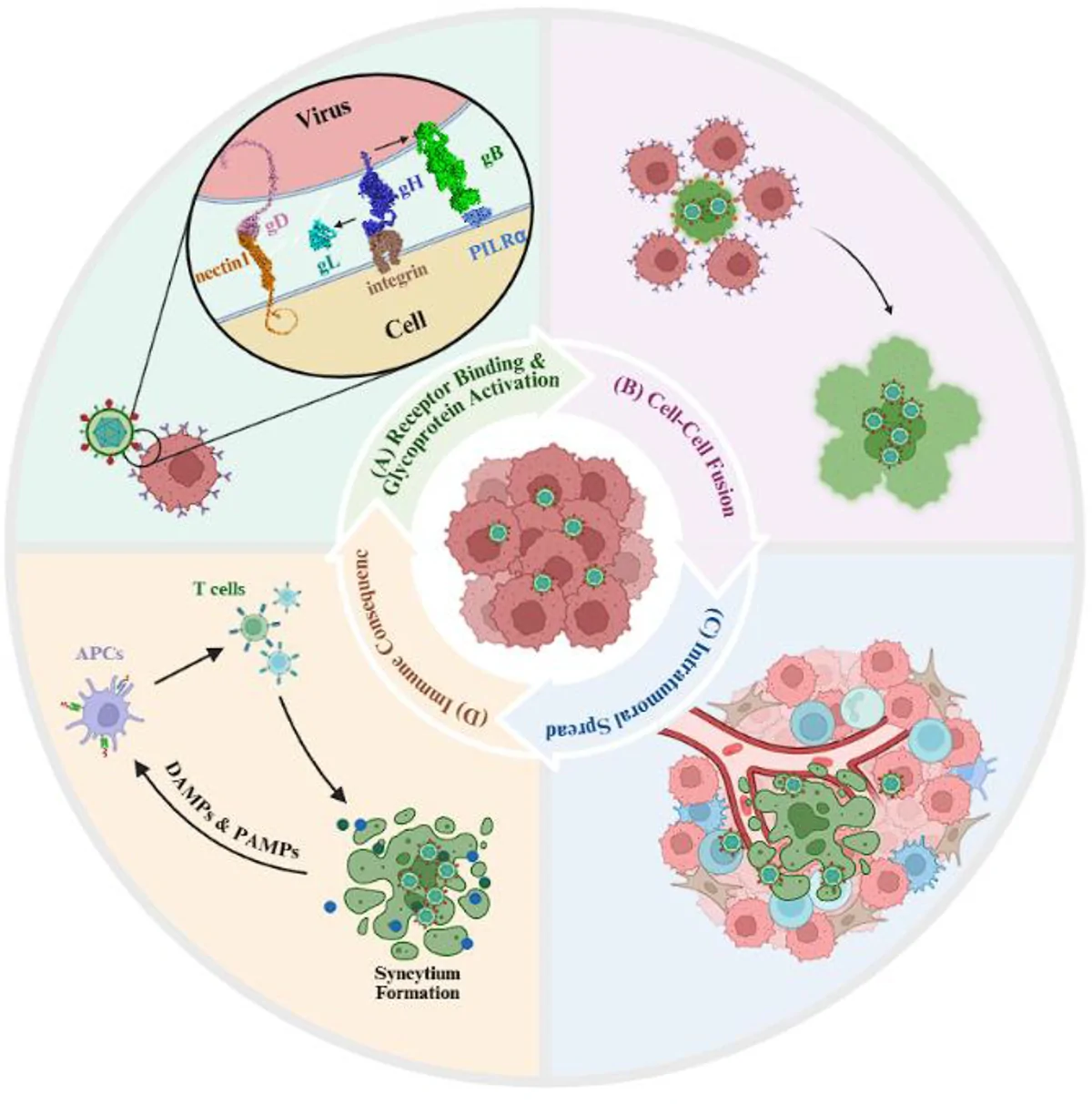
Therapeutic mechanisms and immune consequences of SF-HSV-1 virotherapy. (A) Receptor binding & glycoprotein activation: viral entry is initiated by glycoprotein D (gD) engaging host cellular receptors (e.g. nectin-1). This interaction triggers a sequential signaling cascade through the gH/gL complex to activate the core fusogen, glycoprotein B (gB). (B) Cell-cell fusion: driven by specific genetic determinants, such as mutations in the gB C-terminal domain (CTD) and the gK/UL20 regulatory axis, the viral fusion proteins expressed on the infected cell surface mediate direct fusion with adjacent uninfected tumor cells, forming multinucleated giant cells (syncytia). (C) Intratumoral spread: by traversing the tumor stroma intracellularly, the spreading virions remain completely insulated from pre-existing neutralizing antibodies in the tumor microenvironment. (D) Immune consequences: SF-HSV-1 induces severe cell lysis, thereby promoting ICD in tumor cells. This synchronized collapse releases abundant damage-associated molecular patterns (DAMPs, such as calreticulin, HMGB1, and ATP), which robustly recruit and activate dendritic cells (DCs) to cross-present tumor-associated antigens and prime systemic CD8+ T-cell-mediated antitumor immunity.

Moving forward, the most critical future directions for SF-HSV-1 lie at the convergence of synthetic virology and personalized immunotherapy. Currently, syncytium formation in clinical-stage viruses is exclusively driven by the expression of exogenous genes. Future studies should compare the therapeutic efficacy of SF-HSV-1 induced by inherent viral gene mutations, such as those in *gB*, with those engineered through synthetic biology approaches. Clinically, viewing SF-HSV-1 as a standalone monotherapy is increasingly obsolete. The catastrophic localized ICD and robust DAMP release triggered by syncytial collapse create a highly primed immune niche that must be meticulously coupled with specific immune checkpoint inhibitors (ICIs) or adoptive cellular therapies to overcome T-cell exhaustion and sustain durable systemic remissions. In summary, while SF-HSV-1 platforms remain highly investigational, their dual capacity to mechanically dismantle physical tumor barriers and systemically reprogram the immunosuppressive TME positions them at the vanguard of next-generation cancer therapeutics. Addressing these challenges will ultimately determine whether SF-HSV-1 can serve as a clinically effective extension of current oncolytic HSV-1 therapies (Figure 4).

## Data Availability

No new data were created or analyzed in this study. Data sharing is not applicable to this article.

## References

[1] Suzuki R, Yamasoba D, Kimura I, et al. Attenuated fusogenicity and pathogenicity of SARS-CoV-2 omicron variant. Nature. 2022;603(7902):700–15. doi: 10.1038/s41586-022-04462-1 35104835 PMC8942852

[2] Cesana D, Cicalese MP, Calabria A, et al. A case of T-cell acute lymphoblastic leukemia in retroviral gene therapy for ADA-SCID. Nat Commun. 2024;15(1):3662. doi: 10.1038/s41467-024-47866-5 38688902 PMC11061298

[3] Komoll RM, Hu Q, Olarewaju O, et al. MicroRNA-342-3p is a potent tumour suppressor in hepatocellular carcinoma. J Hepatol. 2021;74(1):122–134. doi: 10.1016/j.jhep.2020.07.039 32738449

[4] Bulcha JT, Wang Y, Ma H, et al. Viral vector platforms within the gene therapy landscape. Signal Trans Targeted Therapy. 2021;6(1):53. doi: 10.1038/s41392-021-00487-6 PMC7868676 33558455

[5] Boorjian SA, Alemozaffar M, Konety BR, et al. Intravesical nadofaragene firadenovec gene therapy for BCG-unresponsive non-muscle-invasive bladder cancer: a single-arm, open-label, repeat-dose clinical trial. Lancet Oncol. 2021;22(1):107–117. doi: 10.1016/s1470-2045(20)30540-4 33253641 PMC7988888

[6] Suchy FP, Karigane D, Nakauchi Y, et al. Genome engineering with Cas9 and AAV repair templates generates frequent concatemeric insertions of viral vectors. Nat Biotechnol. 2025;43(2):204–213. doi: 10.1038/s41587-024-02171-w 38589662 PMC11524221

[7] Mendell JR, Al-Zaidy SA, Rodino-Klapac LR, et al. Current clinical applications of in vivo gene therapy with AAVs. Mol Ther. 2021;29(2):464–488. doi: 10.1016/j.ymthe.2020.12.007 33309881 PMC7854298

[8] Duncan CN, Bledsoe JR, Grzywacz B, et al. Hematologic cancer after gene therapy for cerebral adrenoleukodystrophy. N Engl J Med. 2024;391(14):1287–1301. doi: 10.1056/NEJMoa2405541 39383458 PMC11846662

[9] Ling AL, Solomon IH, Landivar AM, et al. Clinical trial links oncolytic immunoactivation to survival in glioblastoma. Nature. 2023;623(7985):157–166. doi: 10.1038/s41586-023-06623-2 37853118 PMC10620094

[10] Sun L, Funchain P, Song JM, et al. Talimogene Laherparepvec combined with anti-PD-1 based immunotherapy for unresectable stage III-IV melanoma: a case series. J Immunother Cancer. 2018;6(1):36. doi: 10.1186/s40425-018-0337-7 29764498 PMC5954455

[11] Hirooka Y, Kasuya H, Ishikawa T, et al. A Phase I clinical trial of EUS-guided intratumoral injection of the oncolytic virus, HF10 for unresectable locally advanced pancreatic cancer. BMC Cancer. 2018;18(1):596. doi: 10.1186/s12885-018-4453-z 29801474 PMC5970460

[12] Monga V, Miller BJ, Tanas M, et al. Intratumoral talimogene laherparepvec injection with concurrent preoperative radiation in patients with locally advanced soft-tissue sarcoma of the trunk and extremities: phase IB/II trial. J Immunother Cancer. 2021;9(7):e003119. doi: 10.1136/jitc-2021-003119 34330766 PMC8327848

[13] Puzanov I, Milhem MM, Minor D, et al. Talimogene Laherparepvec in combination with ipilimumab in Previously untreated, unresectable stage IIIB-IV melanoma. J Clin Oncol. 2016;34(22):2619–2626. doi: 10.1200/jco.2016.67.1529 27298410 PMC7189507

[14] Andtbacka RH, Ross M, Puzanov I, et al. Patterns of clinical response with Talimogene Laherparepvec (T-VEC) in patients with melanoma treated in the OPTiM Phase III clinical trial. Ann Surg Oncol. 2016;23(13):4169–4177. doi: 10.1245/s10434-016-5286-0 27342831 PMC5090012

[15] Tian Y, Xie D, Yang L. Engineering strategies to enhance oncolytic viruses in cancer immunotherapy. Signal Trans Targeted Therapy. 2022;7(1):117. doi: 10.1038/s41392-022-00951-x PMC8987060 35387984

[16] Nelson A, McMullen N, Gebremeskel S, et al. Fusogenic vesicular stomatitis virus combined with natural killer T cell immunotherapy controls metastatic breast cancer. Breast Cancer Res. 2024;26(1):78. doi: 10.1186/s13058-024-01818-5 38750591 PMC11094881

[17] Feliciano D, Ott CM, Espinosa-Medina I, et al. YAP1 nuclear efflux and transcriptional reprograming follow membrane diminution upon VSV-G-induced cell fusion. Nat Commun. 2021;12(1):4502. doi: 10.1038/s41467-021-24708-2 34301937 PMC8302681

[18] Braga L, Ali H, Secco I, et al. Drugs that inhibit TMEM16 proteins block SARS-CoV-2 spike-induced syncytia. Nature. 2021;594(7861):88–93. doi: 10.1038/s41586-021-03491-6 33827113 PMC7611055

[19] Dai MW, Luo KJ. Envelope-fusion-syncytium formation in microplitis bicoloratus bracovirus maturation. Viruses. 2022;14(10):2183. doi: 10.3390/v14102183 36298738 PMC9608618

[20] Zhou M, Vollmer B, Machala E, et al. Targeted mutagenesis of the herpesvirus fusogen central helix captures transition states. Nat Commun. 2023;14(1):7958. doi: 10.1038/s41467-023-43011-w 38042814 PMC10693595

[21] Fan S, Shen Y, Li S, et al. The S2 subunit of infectious bronchitis virus affects Abl2-mediated syncytium formation. Viruses. 2023;15(6):1246. doi: 10.3390/v15061246 37376546 PMC10301418

[22] Amurri L, Dumont C, Pelissier R, et al. Multifaceted activation of STING axis upon Nipah and measles virus-induced syncytia formation. PLoS Pathog. 2024;20(9):e1012569. doi: 10.1371/journal.ppat.1012569 39283943 PMC11426520

[23] Gianopulos KA, Makio AO, Pritchard SM, et al. Herpes simplex virus 1 glycoprotein B from a hyperfusogenic virus mediates enhanced cell–cell fusion. Viruses. 2024;16(2):251. doi: 10.3390/v16020251 38400027 PMC10892784

[24] Altomonte J, Marozin S, Schmid RM, et al. Engineered newcastle disease virus as an improved oncolytic agent against hepatocellular carcinoma. Mol Ther. 2010;18(2):275–284. doi: 10.1038/mt.2009.231 19809404 PMC2839313

[25] Thomas S, Kuncheria L, Roulstone V, et al. Development of a new fusion-enhanced oncolytic immunotherapy platform based on herpes simplex virus type 1. J Immunother Cancer. 2019;7(1):214. doi: 10.1186/s40425-019-0682-1 31399043 PMC6689178

[26] Wong MKK, Sacco JJ, Robert C, et al. Efficacy and safety of RP1 combined with nivolumab in patients with anti–PD-1–failed melanoma from the IGNYTE clinical trial. J Clin Oncol. 2024;42(16):9517–17. doi: 10.1200/JCO.2024.42.16_suppl.9517

[27] Chmielowski B, Milhem MM, Sacco JJ, et al. Initial efficacy and safety of RP1 + nivolumab in patients with anti–PD-1–failed melanoma from the ongoing phase 1/2 IGNYTE study. J Clin Oncol. 2023;41(16_suppl):9509–09. doi: 10.1200/JCO.2023.41.16_suppl.9509

[28] Schulz KS, Klupp BG, Granzow H, et al. Glycoproteins gB and gH are required for syncytium formation but not for herpesvirus-induced nuclear envelope breakdown. J Virol. 2013;87(17):9733–9741. doi: 10.1128/jvi.01401-13 23824797 PMC3754109

[29] Chouljenko VN, Iyer AV, Chowdhury S, et al. The herpes simplex virus type 1 UL20 protein and the amino terminus of glycoprotein K (gK) physically interact with gB. J Virol. 2010;84(17):8596–8606. doi: 10.1128/jvi.00298-10 20573833 PMC2919038

[30] Kite J, Russell T, Jones J, et al. Cell-to-cell transmission of HSV1 in human keratinocytes in the absence of the major entry receptor, nectin1. PLoS Pathog. 2021;17(9):e1009631. doi: 10.1371/journal.ppat.1009631 34587223 PMC8505007

[31] Nicola AV. Herpesvirus entry into host cells mediated by endosomal low pH. Traffic. 2016;17(9):965–975. doi: 10.1111/tra.12408 27126894 PMC5444542

[32] Jambunathan N, Clark CM, Musarrat F, et al. Two sides to every story: herpes simplex type-1 viral glycoproteins gB, gD, gH/gl, gK, and cellular receptors function as key players in membrane fusion. Viruses-Basel. 2021;13(9):1849. doi: 10.3390/v13091849 PMC8472851 34578430

[33] Pataki Z, Viveros AR, Heldwein EE. Herpes simplex virus 1 entry glycoproteins form complexes before and during membrane fusion. Mbio. 2022;13(5). doi: 10.1128/mbio.02039-22 PMC9600979 35972147

[34] Uchida H, Chan J, Goins WF, et al. A double mutation in glycoprotein gB compensates for ineffective gD-dependent initiation of herpes simplex virus type 1 infection. J Virol. 2010;84(23):12200–12209. doi: 10.1128/jvi.01633-10 20861246 PMC2976401

[35] Gianopulos KA, Sari TK, Weed DJ, et al. Conformational changes in herpes simplex virus glycoprotein C. J Virol. 2022;96(16). doi: 10.1128/jvi.00163-22 PMC9400475 35913218

[36] Nicola AV, McEvoy AM, Straus SE. Roles for endocytosis and low pH in herpes simplex virus entry into HeLa and Chinese hamster ovary cells. J Virol. 2003;77(9):5324–5332. doi: 10.1128/jvi.77.9.5324-5332.2003 12692234 PMC153978

[37] Nicola AV, Straus SE. Cellular and viral requirements for rapid endocytic entry of herpes simplex virus. J Virol. 2004;78(14):7508–7517. doi: 10.1128/jvi.78.14.7508-7517.2004 15220424 PMC434080

[38] Dollery SJ, Wright CC, Johnson DC, et al. Low-pH-dependent changes in the conformation and oligomeric state of the prefusion form of herpes simplex virus glycoprotein B are separable from fusion activity. J Virol. 2011;85(19):9964–9973. doi: 10.1128/jvi.05291-11 21813610 PMC3196434

[39] Sari TK, Pritchard SM, Cunha CW, et al. Contributions of herpes simplex virus 1 envelope proteins to entry by endocytosis. J Virol. 2013;87(24):13922–13926. doi: 10.1128/jvi.02500-13 24109213 PMC3838226

[40] Johnson DC, Huber MT. Directed egress of animal viruses promotes cell-to-cell spread. J Virol. 2002;76(1):1–8. doi: 10.1128/jvi.76.1.1-8.2002 11739666 PMC135733

[41] Johnson DC, Webb M, Wisner TW, et al. Herpes simplex virus gE/gI sorts nascent virions to epithelial cell junctions, promoting virus spread. J Virol. 2001;75(2):821–833. doi: 10.1128/jvi.75.2.821-833.2001 11134295 PMC113978

[42] Dingwell KS, Brunetti CR, Hendricks RL, et al. Herpes simplex virus glycoproteins E and I facilitate cell-to-cell spread in vivo and across junctions of cultured cells. J Virol. 1994;68(2):834–845. doi: 10.1128/jvi.68.2.834-845.1994 8289387 PMC236520

[43] Geraghty RJ, Krummenacher C, Cohen GH, et al. Entry of alphaherpesviruses mediated by poliovirus receptor-related protein 1 and poliovirus receptor. Science. 1998;280(5369):1618–1620. doi: 10.1126/science.280.5369.1618 9616127

[44] Lopez M, Cocchi F, Menotti L, et al. Nectin2α (PRR2α or HveB) and Nectin2δ are low-efficiency mediators for entry of herpes simplex virus mutants carrying the Leu25Pro substitution in glycoprotein D. J Virol. 2000;74(3):1267–1274. doi: 10.1128/jvi.74.3.1267-1274.2000 10627537 PMC111461

[45] Blank H, Burgoon CF, Baldridge GD, et al. Cytologic smears in diagnosis of herpes simplex, herpes zoster, and varicella. J Am Med Assoc. 1951;146(15):1410–1412. doi: 10.1001/jama.1951.63670150005012b 14850308

[46] Ambrosini AE, Enquist LW. Cell-fusion events induced by α-herpesviruses. Future Virol. 2015;10(2):185–200. doi: 10.2217/fvl.14.100

[47] Hussin A, Nor NSM, Ibrahim N. Phenotypic and genotypic characterization of induced acyclovir-resistant clinical isolates of herpes simplex virus type 1. Antiviral Res. 2013;100(2):306–313. doi: 10.1016/j.antiviral.2013.09.008 24055837

[48] Rose L, Crowley B. Molecular characterization of clinical isolates of herpes simplex virus type 1 collected in a tertiary-care hospital in Dublin, Ireland. J Med Virol. 2013;85(5):839–844. doi: 10.1002/jmv.23541 23508909

[49] Carmichael JC, Wills JW. Differential requirements for gE, gI, and UL16 among herpes simplex virus 1 syncytial variants suggest unique modes of dysregulating the mechanism of cell-to-cell spread. J Virol. 2019;93(15). doi: 10.1128/jvi.00494-19 PMC6639296 31092572

[50] Fan Q, Longnecker R, Connolly SA. Herpes simplex virus glycoprotein B mutations define structural sites in domain I, the membrane proximal region, and the cytodomain that regulate entry. J Virol. 2021;95(22). doi: 10.1128/jvi.01050-21 PMC8549500 34431697

[51] Luo Y, Lin C, Ren W, et al. Intravenous injections of a rationally selected oncolytic herpes virus as a potent virotherapy for hepatocellular carcinoma. Mol Ther - Oncolytics. 2019;15:153–165. doi: 10.1016/j.omto.2019.09.004 31720372 PMC6838930

[52] Bertrand L, Leiva-Torres GA, Hyjazie H, et al. Conserved residues in the UL24 protein of herpes simplex virus 1 are important for dispersal of the nucleolar protein nucleolin. J Virol. 2010;84(1):109–118. doi: 10.1128/jvi.01428-09 19864385 PMC2798432

[53] Haanes EJ, Nelson CM, Soule CL, et al. The UL45 gene product is required for herpes simplex virus type 1 glycoprotein B-induced fusion. J Virol. 1994;68(9):5825–5834. doi: 10.1128/jvi.68.9.5825-5834.1994 8057463 PMC236987

[54] Sarfo A, Starkey J, Mellinger E, et al. The UL21 tegument protein of herpes simplex virus 1 is differentially required for the syncytial phenotype. J Virol. 2017;91(21). doi: 10.1128/jvi.01161-17 PMC5640837 28794039

[55] White JM, Delos SE, Brecher M, et al. Structures and mechanisms of viral membrane fusion proteins: multiple variations on a common theme. Crit Rev Biochem Mol Biol. 2008;43(3):189–219. doi: 10.1080/10409230802058320 18568847 PMC2649671

[56] Maurer UE, Zeev-Ben-Mordehai T, Pandurangan AP, et al. The structure of herpesvirus fusion glycoprotein B-bilayer complex reveals the protein-membrane and lateral protein-protein interaction. Structure. 2013;21(8):1396–1405. doi: 10.1016/j.str.2013.05.018 23850455 PMC3737472

[57] Bzik DJ, Fox BA, DeLuca NA, et al. Nucleotide sequence specifying the glycoprotein gene, gB, of herpes simplex virus type 1. Virology. 1984;133(2):301–314. doi: 10.1016/0042-6822(84)90397-0 6324454

[58] Bzik DJ, Fox BA, DeLuca NA, et al. Nucleotide sequence of a region of the herpes simplex virus type 1 gB glycoprotein gene: mutations affecting rate of virus entry and cell fusion. Virology. 1984;137(1):185–190. doi: 10.1016/0042-6822(84)90022-9 6089415

[59] Cai WH, Gu B, Person S. Role of glycoprotein B of herpes simplex virus type 1 in viral entry and cell fusion. J Virol. 1988;62(8):2596–2604. doi: 10.1128/jvi.62.8.2596-2604.1988 2839688 PMC253689

[60] Baghian A, Huang L, Newman S, et al. Truncation of the carboxy-terminal 28 amino acids of glycoprotein B specified by herpes simplex virus type 1 mutant amb1511-7 causes extensive cell fusion. J Virol. 1993;67(4):2396–2401. doi: 10.1128/jvi.67.4.2396-2401.1993 8383250 PMC240410

[61] Lingen M, Seck T, Weise K, et al. Single amino acid substitutions in the glycoprotein B carboxy terminus influence the fusion from without property of herpes simplex virus type 1. J Gen Virol. 1995;76(7):1843–1849. doi: 10.1099/0022-1317-76-7-1843 9049391

[62] Lingen M, Seck T, Weise K, et al. Two mutations in gB-1 and gD-1 of herpes simplex virus type 1 are involved in the ?fusion from without? phenotype in different cell types. Virus Genes. 1996;13(3):221–228. doi: 10.1007/bf00366982 9035366

[63] Silverman JL, Greene NG, King DS, et al. Membrane requirement for folding of the herpes simplex virus 1 gB cytodomain suggests a unique mechanism of fusion regulation. J Virol. 2012;86(15):8171–8184. doi: 10.1128/jvi.00932-12 22623783 PMC3421659

[64] Manservigi R, Spear PG, Buchan A. Cell fusion induced by herpes simplex virus is promoted and suppressed by different viral glycoproteins. Proc Natl Acad Sci USA. 1977;74(9):3913–3917. doi: 10.1073/pnas.74.9.3913 198812 PMC431783

[65] Foster TP, Alvarez X, Kousoulas KG. Plasma membrane topology of syncytial domains of herpes simplex virus type 1 glycoprotein K (gK): the UL20 protein enables cell surface localization of gK but not gK-mediated cell-to-cell fusion. J Virol. 2003;77(1):499–510. doi: 10.1128/jvi.77.1.499-510.2003 12477855 PMC140622

[66] Dolter KE, Ramaswamy R, Holland TC. Syncytial mutations in the herpes simplex virus type 1 gK (UL53) gene occur in two distinct domains. J Virol. 1994;68(12):8277–8281. doi: 10.1128/jvi.68.12.8277-8281.1994 7966620 PMC237295

[67] Pogue-Geile KL, Spear PG. The single base pair substitution responsible for the Syn phenotype of herpes simplex virus type 1, strain MP. Virology. 1987;157(1):67–74. doi: 10.1016/0042-6822(87)90314-x 3029967

[68] Baines JD, Ward PL, Campadelli-Fiume G, et al. The UL20 gene of herpes simplex virus 1 encodes a function necessary for viral egress. J Virol. 1991;65(12):6414–6424. doi: 10.1128/jvi.65.12.6414-6424.1991 1719228 PMC250678

[69] Foster TP, Melancon JM, Baines JD, et al. The herpes simplex virus type 1 UL20 protein modulates membrane fusion events during cytoplasmic virion morphogenesis and virus-induced cell fusion. J Virol. 2004;78(10):5347–5357. doi: 10.1128/jvi.78.10.5347-5357.2004 15113914 PMC400383

[70] Turner A, Bruun B, Minson T, et al. Glycoproteins gB, gD, and gHgL of herpes simplex virus type 1 are necessary and sufficient to mediate membrane fusion in a cos cell transfection system. J Virol. 1998;72(1):873–875. doi: 10.1128/jvi.72.1.873-875.1998 9420303 PMC109452

[71] Avitabile E, Lombardi G, Gianni T, et al. Coexpression of UL20p and gK inhibits cell-cell fusion mediated by herpes simplex virus glycoproteins gD, gH-gL, and wild-type gB or an endocytosis-defective gB mutant and downmodulates their cell surface expression. J Virol. 2004;78(15):8015–8025. doi: 10.1128/jvi.78.15.8015-8025.2004 15254173 PMC446093

[72] Rauch DA, Rodriguez N, Roller RJ. Mutations in herpes simplex virus glycoprotein D distinguish entry of free virus from cell-cell spread. J Virol. 2000;74(24):11437–11446. doi: 10.1128/jvi.74.24.11437-11446.2000 11090139 PMC112422

[73] Pearson A, Coen DM. Identification, localization, and regulation of expression of the UL24 protein of herpes simplex virus type 1. J Virol. 2002;76(21):10821–10828. doi: 10.1128/jvi.76.21.10821-10828.2002 12368325 PMC136619

[74] Bryant KF, Macari ER, Malik N, et al. ICP34.5-dependent and -independent activities of salubrinal in herpes simplex virus-1 infected cells. Virology. 2008;379(2):197–204. doi: 10.1016/j.virol.2008.06.028 18684481 PMC2665023

[75] Carmichael JC, Yokota H, Craven RC, et al. The HSV-1 mechanisms of cell-to-cell spread and fusion are critically dependent on host PTP1B. PLoS Pathog. 2018;14(5):e1007054. doi: 10.1371/journal.ppat.1007054 29742155 PMC5962101

[76] Saito A, Irie T, Suzuki R, et al. Enhanced fusogenicity and pathogenicity of SARS-CoV-2 delta P681R mutation. Nature. 2022;602(7896):300–306. doi: 10.1038/s41586-021-04266-9 34823256 PMC8828475

[77] Burton C, Bartee E. Syncytia formation in oncolytic virotherapy. Mol Ther - Oncolytics. 2019;15:131–139. doi: 10.1016/j.omto.2019.09.006 31890866 PMC6931088

[78] Prince AM, Ginsberg HS. Studies on the cytotoxic effect of Newcastle disease virus (NDV) on Ehrlich ascites tumor cells. I. Characteristics of the virus-cell interaction. J Immunol (Baltim, Md : 1950). 1957;79(2):94–106. doi: 10.4049/jimmunol.79.2.94 13475814

[79] Freeman AI, Zakay-Rones Z, Gomori JM, et al. Phase I/II trial of intravenous NDV-HUJ oncolytic virus in recurrent glioblastoma multiforme. Mol Ther. 2006;13(1):221–228. doi: 10.1016/j.ymthe.2005.08.016 16257582

[80] Choi SH, Park BK, Lee K-W, et al. Effect of respiratory syncytial virus on the growth of hepatocellular carcinoma cell-lines. BMB Rep. 2015;48(10):565–570. doi: 10.5483/BMBRep.2015.48.10.268 25739391 PMC4911183

[81] Salimi V, Tavakoli-Yaraki M, Mahmoodi M, et al. The oncolytic effect of respiratory syncytial virus (RSV) in human skin cancer cell line, A431. Iran Red Crescent Med J. 2013;15(1):62–67. doi: 10.5812/ircmj.4722 23487261 PMC3589781

[82] Israyelyan A, Chouljenko VN, Baghian A, et al. Herpes simplex virus type-1(HSV-1) oncolytic and highly fusogenic mutants carrying the NV1020 genomic deletion effectively inhibit primary and metastatic tumors in mice. Virol J. 2008;5(1). doi: 10.1186/1743-422x-5-68 PMC2453120 18518998

[83] Takaoka H, Takahashi G, Ogawa F, et al. A novel fusogenic herpes simplex virus for oncolytic virotherapy of squamous cell carcinoma. Virol J. 2011;8(1). doi: 10.1186/1743-422x-8-294 PMC3131258 21663640

[84] Simpson GR, Han Z, Liu B, et al. Combination of a fusogenic glycoprotein, prodrug activation, and oncolytic herpes simplex virus for enhanced local tumor control. Cancer Res. 2006;66(9):4835–4842. doi: 10.1158/0008-5472.Can-05-4352 16651439

[85] Bing Z, Jianru Y, Yuequan J, et al. Packaging, amplification, and appraisal of the recombinant tumor-selective type I herpes simplex virus carrying GALV.Fus gene. Cell Biochem Biophys. 2014;70(1):321–326. doi: 10.1007/s12013-014-9915-6 24687598

[86] Simpson GR, Horvath A, Annels NE, et al. Combination of a fusogenic glycoprotein, pro-drug activation and oncolytic HSV as an intravesical therapy for superficial bladder cancer. Br J Cancer. 2012;106(3):496–507. doi: 10.1038/bjc.2011.577 22240799 PMC3273343

[87] Xu B, Ma R, Russell L, et al. An oncolytic herpesvirus expressing E-cadherin improves survival in mouse models of glioblastoma. Nat Biotechnol. 2018;37(1):45–54. doi: 10.1038/nbt.4302 PMC6535376 30475349

[88] Zhu B, Yang JR, Fu XP, et al. Anti-tumor effects of gene therapy with GALV membrane fusion glycoprotein in lung adenocarcinoma. Cell Biochem Biophys. 2014;69(3):577–582. doi: 10.1007/s12013-014-9835-5 24519667

[89] Epstein AL, Rabkin SD. Safety of non-replicative and oncolytic replication-selective HSV vectors. Trends Mol Med. 2024;30(8):781–794. doi: 10.1016/j.molmed.2024.05.014 38886138 PMC11329358

[90] Aravalli RN, Hu SX, Rowen TN, et al. Cutting edge: TLR2-mediated proinflammatory cytokine and chemokine production by microglial cells in response to herpes simplex virus. The J Immunol. 2005;175(7):4189–4193. doi: 10.4049/jimmunol.175.7.4189 16177057

[91] Kurt-Jones EA, Chan M, Zhou SH, et al. Herpes simplex virus 1 interaction with Toll-like receptor 2 contributes to lethal encephalitis. Proc Natl Acad Sci USA. 2004;101(5):1315–1320. doi: 10.1073/pnas.0308057100 14739339 PMC337050

[92] Sato R, Kato A, Chimura T, et al. Combating herpesvirus encephalitis by potentiating a TLR3–mTORC2 axis. Nat Immunol. 2018;19(10):1071–1082. doi: 10.1038/s41590-018-0203-2 30201994

[93] O’Connor CM, Sen GC. Innate immune responses to herpesvirus infection. Cells. 2021;10(8):2122. doi: 10.3390/cells10082122 34440891 PMC8394705

[94] Carpenter S, O’Neill LAJ. From periphery to center stage: 50 years of advancements in innate immunity. Cell. 2024;187(9):2030–2051. doi: 10.1016/j.cell.2024.03.036 38670064 PMC11060700

[95] Choi MK, Wang Z, Ban T, et al. A selective contribution of the RIG-I-like receptor pathway to type I interferon responses activated by cytosolic DNA. Proc Natl Acad Sci USA. 2009;106(42):17870–17875. doi: 10.1073/pnas.0909545106 19805092 PMC2764914

[96] Chiang JJ, Sparrer KMJ, van Gent M, et al. Viral unmasking of cellular 5S rRNA pseudogene transcripts induces RIG-I-mediated immunity. Nat Immunol. 2018;19(1):53–62. doi: 10.1038/s41590-017-0005-y 29180807 PMC5815369

[97] Ishikawa H, Ma Z, Barber GN. STING regulates intracellular DNA-mediated, type I interferon-dependent innate immunity. Nature. 2009;461(7265):788–792. doi: 10.1038/nature08476 19776740 PMC4664154

[98] Sun L, Wu J, Du F, et al. Cyclic GMP-AMP synthase is a cytosolic DNA sensor that activates the type I interferon pathway. Science. 2013;339(6121):786–791. doi: 10.1126/science.1232458 23258413 PMC3863629

[99] Wang C, Wang X, Veleeparambil M, et al. EGFR-mediated tyrosine phosphorylation ofStingdetermines its trafficking route and cellular innate immunity functions. Embo J. 2020;39(22). doi: 10.15252/embj.2019104106 PMC7667877 32926474

[100] Gram AM, Frenkel J, Ressing ME. Inflammasomes and viruses: cellular defence versus viral offence. J Gen Virol. 2012;93(10):2063–2075. doi: 10.1099/vir.0.042978-0 22739062

[101] Barnett KC, Li S, Liang K, et al. A 360° view of the inflammasome: mechanisms of activation, cell death, and diseases. Cell. 2023;186(11):2288–2312. doi: 10.1016/j.cell.2023.04.025 37236155 PMC10228754

[102] St Leger AJ, Koelle DM, Kinchington PR, et al. Local immune control of latent herpes simplex virus type 1 in ganglia of mice and man. Front Immunol. 2021;12. doi: 10.3389/fimmu.2021.723809 PMC8479180 34603296

[103] Berber E, Rouse BT. Controlling herpes simplex virus-induced immunoinflammatory lesions using metabolic therapy: a comparison of 2-deoxy-d-glucose with metformin. J Virol. 2022;96(14). doi: 10.1128/jvi.00688-22 PMC9327707 35862706

[104] Bateman AR, Harrington KJ, Kottke T, et al. Viral fusogenic membrane glycoproteins kill solid tumor cells by nonapoptotic mechanisms that promote cross presentation of tumor antigens by dendritic cells. Cancer Res. 2002;62(22):6566–6578. 12438252

[105] Hoffmann D, Bayer W, Wildner O. Therapeutic immune response induced by intratumoral expression of the fusogenic membrane protein of vesicular stomatitis virus and cytokines encoded by adenoviral vectors. Int J Mol Med. 2007;20(5):673–681. 17912460

[106] Errington F, Jones J, Merrick A, et al. Fusogenic membrane glycoprotein-mediated tumour cell fusion activates human dendritic cells for enhanced IL-12 production and T-cell priming. Gene Ther. 2006;13(2):138–149. doi: 10.1038/sj.gt.3302609 16136162

[107] Mazzacurati L, Marzulli M, Reinhart B, et al. Use of miRNA response sequences to block off-target replication and increase the safety of an unattenuated, glioblastoma-targeted oncolytic HSV. Mol Ther. 2015;23(1):99–107. doi: 10.1038/mt.2014.177 25200130 PMC4426800

[108] Ylösmäki E, Lavilla-Alonso S, Jäämaa S, et al. MicroRNA-mediated suppression of oncolytic adenovirus replication in human liver. PLOS ONE. 2013;8(1):e54506. doi: 10.1371/journal.pone.0054506 23349911 PMC3551754

[109] Okubo Y, Uchida H, Wakata A, et al. Syncytial mutations do not impair the specificity of entry and spread of a glycoprotein D receptor-retargeted herpes simplex virus. J Virol. 2016;90(24):11096–11105. doi: 10.1128/jvi.01456-16 27707922 PMC5126368

[110] Uchida H, Marzulli M, Nakano K, et al. Effective treatment of an orthotopic xenograft model of human glioblastoma using an EGFR-retargeted oncolytic herpes simplex virus. Mol Ther. 2013;21(3):561–569. doi: 10.1038/mt.2012.211 23070115 PMC3589172

[111] Goins WF, Hall B, Cohen JB, et al. Retargeting of herpes simplex virus (HSV) vectors. Curr Opin Virol. 2016;21:93–101. doi: 10.1016/j.coviro.2016.08.007 27614209 PMC5138136

[112] Xie Z, Wroblewska L, Prochazka L, et al. Multi-input RNAi-based logic circuit for identification of specific cancer cells. Science. 2011;333(6047):1307–1311. doi: 10.1126/science.1205527 21885784

[113] Jiang Y, Sun S, Quan Y, et al. Nuclear RPSA senses viral nucleic acids to promote the innate inflammatory response. Nat Commun. 2023;14(1):8455. doi: 10.1038/s41467-023-43784-0 38114488 PMC10730619

[114] Di Stasi A, Tey SK, Dotti G, et al. Inducible apoptosis as a safety switch for adoptive cell therapy. N Engl J Med. 2011;365(18):1673–1683. doi: 10.1056/NEJMoa1106152 22047558 PMC3236370

[115] Maurer UE, Sodeik B, Grünewald K. Native 3D intermediates of membrane fusion in herpes simplex virus 1 entry. Proc Natl Acad Sci USA. 2008;105(30):10559–10564. doi: 10.1073/pnas.0801674105 18653756 PMC2492464

